# Creating an Indigenous Māori‐centred model of relational health: A literature review of Māori models of health

**DOI:** 10.1111/jocn.15859

**Published:** 2021-05-27

**Authors:** Denise Wilson, Eleanor Moloney, Jenny M. Parr, Cathleen Aspinall, Julia Slark

**Affiliations:** ^1^ Taupua Waiora Māori Research Centre Faculty of Health & Environmental Sciences Auckland University of Technology Manukau, Auckland New Zealand; ^2^ School of Nursing University of Auckland Grafton, Auckland New Zealand; ^3^ Counties Manukau Health District Health Board Middlemore Hospital Auckland New Zealand; ^4^ Counties Manukau Health Otahuhu, Auckland New Zealand

**Keywords:** cultural competency, cultural safety, fundamental care, Indigenous, literature review, nurse‐patient relationship

## Abstract

**Aims and objectives:**

Identify the key concepts, principles and values embedded within Indigenous Māori models of health and wellbeing; and determine how these could inform the development of a Māori‐centred relational model of care.

**Background:**

Improving health equity for Māori, similar to other colonised Indigenous peoples globally, requires urgent attention. Improving the quality of health practitioners’ engagement with Indigenous Māori accessing health services is one area that could support improving Māori health equity. While the Fundamentals of Care framework offers a promising relational approach, it lacks consideration of culture, whānau or family, and spirituality, important for Indigenous health and wellbeing.

**Design and methods:**

A qualitative literature review on Māori models of health and wellbeing yielded nine models to inform a Māori‐centred relational model of care. We followed the PRISMA guidelines for reporting literature reviews.

**Results:**

Four overarching themes were identified that included dimensions of health and wellbeing; whanaungatanga (connectedness); whakawhanaungatanga (building relationships); and socio‐political health context (colonisation, urbanisation, racism, and marginalisation). Health and wellbeing for Māori is a holistic and relational concept. Building relationships that include whānau (extended family) is a cultural imperative.

**Conclusions:**

This study highlights the importance and relevance of relational approaches to engaging Māori and their whānau accessing health services. It signals the necessary foundations for health practitioners to build trust‐based relationships with Māori. Key elements for a Māori‐centred model of relational care include whakawhanaungatanga (the process of building relationships) using tikanga (cultural protocols and processes) informed by cultural values of aroha (compassion and empathy), manaakitanga (kindness and hospitality), mauri (binding energy), wairua (importance of spiritual wellbeing).

**Relevance to clinical practice:**

Culturally‐based models of health and wellbeing provide indicators of important cultural values, concepts and practices and processes. These can then inform the development of a Māori‐centred relational model of care to address inequity.

**What does this paper contribute to the wider global community?**

- Culturally relevant and meaningful approaches to health care service delivery are critical for improving equity in Indigenous and other ethnic groups.
- Relational approaches to engaging with Indigenous peoples and their families are cultural imperatives for accessing health services.
- Culturally‐based health and wellbeing models provide important cultural values, concepts, practices and processes essential for improving health outcomes.

## 1 INTRODUCTION

An issue plaguing Aotearoa New Zealand's (Aotearoa NZ) health system is the continued inability to achieve health equity for the Indigenous Māori population. Many would argue this constitutes not only a health crisis but also provides an ongoing source of shame for the New Zealand government (Waitangi Tribunal, 2019). This persistent inequity is like the poor health outcomes for Indigenous populations globally, especially in settler‐colonial nations such as Australia, Canada, Aotearoa and the United States (Deeble et al., 2008; Gao et al., 2008; Zuckerman et al., 2004).

However, Te Tiriti o Waitangi (a treaty outlining the relationship between Māori and the British Crown that affirmed the rights of Māori – referred to as ‘Te Tiriti’) sets Aotearoa NZ apart from other colonised nations. Te Tiriti is Aotearoa NZ's founding document, signed by the British Crown representatives and many of the Māori chiefs on 6 February 1840. Te Tiriti paved the way for the establishment of the colonial state of New Zealand. Interpretation of the articles of Te Tiriti is a source of ongoing debate, particularly regarding the inconsistencies between the te reo Māori (Māori language) and English versions (referred to as the Treaty of Waitangi) (Mulholland & Tawhai, 2010; Wilson & Haretuku, 2015). While the intent and interpretation of the two versions of Te Tiriti differ, Article Three affirms Māori rights to equity (Wilson & Haretuku, 2015).

### 1.1 Enduring inequities

Despite Māori surviving a challenging and adverse history, ongoing colonisation and its effects significantly impact contemporary Māori (Kingi et al., 2017). Health outcomes confirm the inequitable access many Māori have to the social determinants of health, health services, and safe quality health care (Ministry of Health, 2015). Walsh and Grey (2019) found avoidable mortality explained 53% of the seven‐year life expectancy gap for Māori and is evidence of the lack of access for Māori to timely, safe, and quality health services. The World Health Organization (WHO) described the social determinants of health, including timely access to healthcare, quality education, optimal working conditions, the ability to engage in leisure activities, quality and safe housing and communities (World Health Organization, 2019). According to the WHO, inequities in access to the social determinants of health are not natural phenomena. Inequities in social determinants stem from ineffectual or inadequate political leadership, social policy, and economic structures that serve those with higher socioeconomic status at the expense of those groups of people with low socioeconomic status.

Māori have inequitable experiences in Aotearoa NZ's publicly funded healthcare system, including amenable health determinants such as racism. Research confirms Māori are more likely than other groups in Aotearoa NZ to encounter structural, cultural, and interpersonal forms of discrimination, marginalisation, and racism when accessing healthcare services (Cormack et al., 2018; Wepa & Wilson, 2019; Wilson & Barton, 2012). These negative experiences, such as being treated differently to others and deficit stereotypes and explanations informing health practitioners’ interactions, impact trust and the decisions Māori make about future access to healthcare services (Cormack et al., 2018; Harris et al., 2012a, 2012b; Wilson & Barton, 2012). Compared to non‐Māori, Māori receive poorer quality care when they access healthcare services (Rumball‐Smith et al., 2013) and are more likely to encounter adverse events (Davis et al., 2006). Māori are also less likely to be referred to specialist services, less likely to be prescribed effective medication or surgical interventions, more likely to be discharged from hospital earlier, and more likely to die from amenable diseases (Ellison‐Loschmann & Pearce, 2006; Rumball‐Smith & Hider, 2009; Rumball‐Smith et al., 2013; Wilson & Barton, 2012). Unsurprisingly, Māori experience more disease, metabolic disorders, mental illness, maternal health complications and substance abuse issues than other groups (Wilson & Neville, ).

A systematic review of Māori experiences of the public health system in Aotearoa NZ found institutional level barriers, mainly racism and discrimination (Graham & Masters‐Awatere, 2020; Wilson & Barton, 2012). Further, the systematic review reported Māori encountered unhelpful healthcare practitioners with negative attitudes. Māori also encountered other barriers related to costs, transport, and organisation of daily lives to access healthcare services (Graham & Masters‐Awatere, 2020). However, Graham and Masters‐Awatere highlighted the value of whānau support in helping Māori to navigate the healthcare system and provide practical and emotional support. Nonetheless, research with Māori and their whānau found healthcare environments were generally unfriendly and culturally foreign (Wilson & Barton, 2012; Anderson & Spray, 2020). Māori whānau also reported not knowing the rules for engagement with healthcare providers (Wepa & Wilson, 2019). Māori and their whānau often encountered healthcare practitioners who were hostile and disrespectful in their attitudes and behaviours and ineffective in the care they provided. This left Māori whānau feeling marginalised (Wilson & Barton, 2012) and silenced (Wepa & Wilson, 2019) and impacting the quality of services they received. Such interactions invariably led to Māori and whānau reporting sub‐standard care and not having their healthcare needs met in part or fully. The outcomes of such encounters affected trust, the ability to navigate a complex health system and led to avoidance (Wepa & Wilson, 2019; Wilson & Barton, 2012). As Wepa and Wilson stated, "They survived by having hope, feeling lucky and taking control of their healthcare journey" (p.3).

The Waitangi Tribunal (2019) inquiry into primary healthcare services and outcomes for Māori (the ‘WAI2575 report’) found detrimental colonial structures functioned in Aotearoa New Zealand's healthcare system. The Waitangi Tribunal highlighted the government's continued inconsistencies in applying the principles of the Treaty, "partnership, active protection and equity" (p. 162), and failed to honour its obligations to provide equitable health outcomes for Māori (Waitangi Tribunal, 2019). The health care system in Aotearoa NZ, is informed primarily by an individualistic, problem‐based, and biomedical approach (HQSC, 2019). Such a worldview contradicts a Māori holistic and relational‐based worldview of health and wellbeing (Jansen et al., 2008; Willams et al., 2003; Wilson & Barton, 2012). Māori reported hospital‐based health care experiences as stressful and alienating that engendered anxiety and discomfort for people and their whānau (extended family network) (Wepa & Wilson, 2019; Wilson & Barton, 2012). For the most part, Māori experience hospital settings not as healing environments but as a place where their cultural and spiritual beliefs and practices were dismissed or not considered. Instead, health services specifically catered to the needs of those belonging to the dominant New Zealand European culture (Wilson & Barton, 2012). The privileging of a western medical worldview and the culturally unsafe environment that creates also impacts the recruitment and retention of Māori health practitioners, evident in the inequity and under‐representation of Māori in the health workforce (Sewell, 2017).

### 1.2 Fundamentals of Care

The Fundamentals of Care Framework is being implemented by nurses in Aotearoa to optimise patient‐centred care and the quality of nurses’ interactions with patients (Aspinall et al., 2020). Several health organisations have implemented a unit‐level quality measurement and improvement programme to be assured of the extent of delivery of fundamental care and make the contribution of nursing visible (Parr et al., 2018). The Fundamentals of Care framework was developed by the International Learning Collaborative (ILC) following the Francis Inquiry in England which demonstrated failures of delivery of seemingly basic care by nurses (Kitson et al., 2013). Its purpose was to ensure the patient's voice was heard within the health system, to provide visibility of the contribution and importance of nursing care and align contextual factors to the nature of nursing care. Nursing care is in fact anything but basic (Kitson et al., 2014), and the framework articulates this complexity when providing patient focussed integrated care, within the context of the care setting. A notable omission in the current Fundamentals of Care framework is culture. Research indicates cultural understandings of people's health and wellbeing can significantly impact equitable access to healthcare for Māori (Wepa & Wilson, 2019; Wilson, 2008). Therefore, the Fundamentals of Care framework requires consideration of the person's cultural beliefs and practices when establishing the nurse‐patient relationship and subsequent care provision (Aspinall et al., 2020).

The Fundamentals of Care framework provides a theoretical relational framework to underpin nurses’ everyday practice, guiding them in delivering high quality, patient‐centred care (Kitson, 2018). Central to the Fundamentals of Care framework is forming a robust therapeutic relationship between the nurse and patient. The therapeutic relationship enables the nurse and patient to work together to meet the patient's physical, psychosocial, and relational needs while responding appropriately to specific healthcare environments’ challenges (Kitson, 2018). According to Kitson (2018), establishing a strong therapeutic relationship with a patient signals a commitment to care and requires five elements: building trust, providing undivided attention to the patient, anticipating the patient's needs, knowing the patient well enough to be able to respond appropriately to their care needs, and being able to evaluate the quality of the relationship (Kitson, 2018).

The relational context of the Fundamentals of Care framework offers potential given the cultural imperative of relationships for Māori. Māori worldviews are holistic and require consideration of not only people's physical and psychosocial, but importantly, their relational needs. However, for equitable implementation of the Fundamentals of Care framework within Aotearoa NZ, this relational model of care needs to be Māori‐centred, informed by Māori principles and values.

Change is required in the dominant healthcare system to honour Te Tiriti obligations and achieve equity. Such change requires inclusive healthcare services that embrace Māori ways of thinking and being. The key to improving health equity for Māori is addressing the under‐representation of the Māori workforce and enabling Māori healthcare practitioners to provide culturally grounded care for Māori (Kingi et al., 2017; Theunissen, 2011). However, achieving the goal of sufficient Māori representation in the workforce will take significant time and commitment. In the meantime, Māori continues to be at risk of accessing sub‐optimal and culturally inadequate healthcare, predominantly provided by a non‐Indigenous workforce.

Fundamental to improving health equity and the quality of healthcare interactions with Māori is establishing respectful relationships with Māori and their whānau. For this paper, relational care refers to the deliberate nurturing of respectful and meaningful relationships with Māori and their whānau. Relational care is a person‐ and whānau‐centred holistic healthcare practice that evolves through mindful reflection and deliberation (Forsyth, 2017; Pohatu, 2013; Wilson, 2008). A range of Māori models and frameworks exist in the health literature. These have been developed for use in particular contexts that may focus on relational aspects of care embedded within them.

## 2 METHODS

### 2.1 Research questions and aims

Two questions guided this literature review: Firstly, what Maori models and frameworks of health and healthcare exist? Secondly, what are the core cultural concepts enveloped in those models? The overall aims of this literature review were, therefore, to: Identify published Māori models and frameworks of health and healthcare, and extract and collate the relevant knowledge embedded within these models; and synthesise and distil the core cultural concepts, values, or principles present in the models and frameworks to inform the development of a Māori centred relational care model for future implementation and testing.

Literature reviews enable broader, more exploratory research aims (Grant & Booth, 2009). This allows mapping the key concepts that underpin a field of research, defining the conceptual boundaries of a topic, and clarifying key concepts and definitions thematically within the literature on a specific issue (Arksey & O'Malley, 2005). This literature review goes beyond a scoping review because it will inform clinical practice. The review followed the framework outlined by Arksey and O'Malley (2005) because it provided a systematic framework and process to review the literature. Therefore a literature review methodology was the most appropriate to examine the current literature (that may or may not include research findings) to identity, map, and define critical cultural concepts, values, and principles within Māori models of health. We followed PRISMA guidelines in undertaking this literature review (File S1).

This literature review focussed on core cultural concepts in Māori models of health pertinent to relational care. Arksey and O'Malley recommended literature reviews be iteratively requiring reflexive engagement to ensure comprehensive coverage of the relevant literature. The methodological framework has five stages: (1) identification of the research question; (2) identification of relevant studies; (3) literature (study) selection; (4) charting the data; and (5) collation, summarising, and reporting the results. The following sub‐sections outline the explication of the literature review methodology undertaken:

### 2.2 Identification of relevant studies

A research librarian supported developing the search strategy to identify relevant literature and search the following four databases: CINAHL Plus, PubMed, SCOPUS, and INDEX New Zealand. We also accessed grey literature using the nzreserch.org database. The following keywords and search terms used included: Māori AND ("model* of health" OR "health model*" OR "model* of care" OR "care model*" OR "framework* of health" OR "health framework*" OR "framework* of care" OR "care framework*"). Using the * symbol to identify either singular or plural forms of the search terms was incompatible with the INDEX New Zealand or nzresearch.org databases, therefore singular forms of the search terms were used as follows: Māori AND ("model of health" OR "health model" OR "model of care" OR "care model" OR "framework of health" OR "health framework" OR "framework of care" OR "care framework"), and then repeated using the plural forms of the search terms as follows: Māori AND ("models of health" OR "health models" OR "models of care" OR "care models" OR "frameworks of health" OR "health frameworks" OR "frameworks of care" OR "care frameworks").

The inclusion criteria for selecting studies included:

- Literature needed to outline a unique Māori model or framework of health or care. More specifically, each piece of literature was required to refer to a model or framework that focussed on either Māori ways of conceptualising health or the relationship between Māori patients and healthcare practitioners.
- No timeframe was applied because seminal texts included models published in the 1990s. For instance, Mason Durie's Te Whare Tapa Whā model (Durie, 1998) and Rose Pere's Te Wheke model (Pere, 1991) are seminal texts widely utilised and cited in the Aotearoa NZ healthcare sector. Moreover, mātauranga Māori (ancient or traditional knowledge) passed down over generations forms the basis of Māori models of health and care frameworks. Therefore, from an Indigenous perspective, models or frameworks published further in the past are not necessarily less relevant than those published more recently.
- Only papers written in English were eligible for inclusion.
- Both peer‐reviewed and non‐peer‐reviewed papers were eligible for inclusion in this review to ensure the inclusion of all models and frameworks available.

Exclusion of literature included models or frameworks that focussed on specific areas of Māori health, such as those that looked at health promotion or system‐level factors, because this literature review aimed to inform the development of a Māori‐centred relational model of care.

### 2.3 Literature selection

**FIGURE 1:**
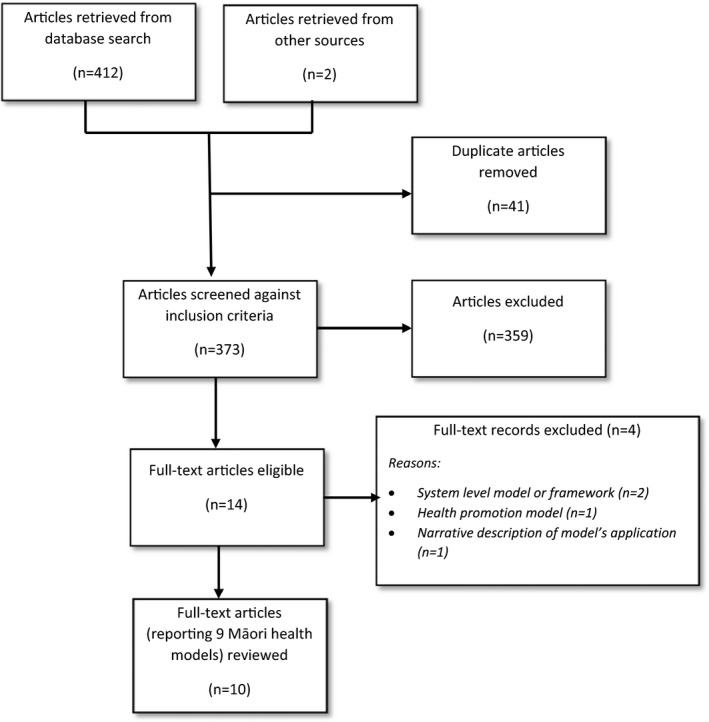
Overview of literature selection

Arksey and O'Malley (2005) recommended using two reviewers to independently screen all citations against the inclusion and exclusion criteria to select the literature for inclusion in the review. First, the reviewer (EM) screened titles and abstracts of retrieved documents against the inclusion criteria. We then retrieved full‐text documents based on the abstracts that met the inclusion criteria. A second reviewer (DW) then reviewed each full‐text paper and together both reviewers decided to include or exclude papers from the final review. This process of reviewers independently reviewing the articles and then coming together to reach a consensus occurred throughout the analytic process. The database search identified 412 records. The reference lists of included documents provided a further two records. After screening the title and abstracts and removing duplicates, we retrieved 14 full‐text documents. Four of these documents were then excluded based on the inclusion criteria, leaving ten articles about Māori health and wellbeing models (see Figure 1).

### 2.4 Charting the data

We extracted narrative data from each paper (by reviewer EM). This included: author/s, the date and source of publication; the context for the model (for example, everyday life, hospital or clinical settings); the target population for the use of the model (for example, all people, doctors, nurses, all healthcare practitioners); the discreet dimensions for each model or framework mapped according to the aims of the review.

### 2.5 Collation, synthesising, and reporting of results

The relationships between the dimensions were collated, sorted and summarised into key themes that captured the underlying cultural and relational concepts and values (Arksey & O'Malley, 2005). The data synthesis included taking the aggregated data mapped according to the literature review's purposes, which were then thematically sorted and labelled to enable a narrative description. The themes reflected the relational aspects and dimensions necessary for a Māori model of relational care. During this process, the reviewers (EM and DW) conferred and agreed on the key themes to report narratively.

## 3 RESULTS

Ten articles describing nine different Māori health models met the criteria for inclusion from 14 articles. Each of these Māori health models provided cultural concepts and principles for use in healthcare practice. Five focussed on optimising the engagement of Māori with health services, while four focussed on Māori perspectives of health and wellbeing (see Table 1). While all models and frameworks varied in their presentation, similarities were also evident. Four overarching themes captured the core cultural concepts and values that would inform the development of a Māori‐centred relational model of care (see Table 2). These themes were: (1) Dimensions of health and wellbeing; (2) Whanaungatanga (connectedness); (3) Whakawhanaugatanga (building relationships); and (4) Socio‐political health context. Each of these themes is intertwined and underpinned by critical Māori values and concepts. These values and concepts play an integral role in the holistic and relational Māori ontology that influence health and wellbeing, connectedness, and building relationships. All of these occur within a socio‐political context for Māori health and wellbeing that feature colonisation, racism, and marginalisation (see Table 2). Table 3 provides a narrative overview of the findings.

**TABLE 1:** Summary of the values, beliefs and concepts within Māori health and wellbeing models

Model	Culturally‐based analogy	Relational focus	Key core values and concepts
Hui Process^1^	Uses hui (meeting, gathering), a culturally‐based formal engagement process to explain the tikanga (right protocol) required for health practitioners and Māori and whānau engagement.	*Engagement*: Uses the hui process that begins with mihi (greeting) and whakawhanaungatanga to forming connections to build relationship. Improving engagement, mutual understanding, and building rapport through cultural competency.	Mihi (initial greeting) Whakawhanaungatanga (making connections that includes whenua, whānau, te reo Māori) Kaupapa (purpose of the encounter) Poroporaki/whakamutunga (closing session)
Kapakapa Manawa Framework^2^	Manawa refers to the heart and kapakapa is defined as causing the heart to beat, throb, pulsate, or flutter. This beating or pulsating of the manawa is said to motivate a person to engage their heartfelt emotions so that they may care for a dying person in meaningful ways.	*Engagement*: Extends a compassionate care framework to encompass Māori values. Aims to provide culturally informed compassionate care and meet the unique needs of Māori and their whānau at the end of life.	He ngākau aroha (expressing care, kind heartedness, consideration for others, compassion and empathy) Whakawhanaungatanga (nurturing ongoing connections by relating well to others) Te tuakirio ngā tangata Māori (knowing the person and using this knowledge to adapt practice) Manaakitanga (reciprocity for sharing and caring for one another and showing mutual respect)
Meihana Model^3^	Extends on the hui process by explicating how the kaupapa part can be used to collect data using the waka hourua (double hulled canoe) to describe navigating the waters, and the four winds of colonisation migration, and racism, marginalisation. The waka hourua highlights how Māori are integrally connected to their whānau.	*Engagement*: Focus is on forming a positive relationship to attain hauora (health and wellbeing) while providing the various aspects that need to be considered when engaging with Māori. Beyond the initial engagement the Meihana model also includes Ngā hau e wha (colonisation, migration, racism, and marginalisation) describes the four winds that impact Māori; and Ngā roma moana (ocean currents – ahua (personal indicators), tikanga, whenua, whānau) that influence navigation of the engagement with Māori.	Whānau (support for person and maybe either whakapapa (genealogical) or kaupapa (other key support people)) Tinana (body) Hinengaro (mind) Wairua (spirit) Taiao (providing a welcoming physical environment) Iwi katoa (services and person systems that provide support)
Te Hā o Whānau^4^	The author defines Te Hā o Whānau as ‘whānay voices leading maternity care’. The model is based around three Māoir tikanga – Manaakitanga (protecting and uplifting mana), whakawhanaungatanga (relationships with people and space), and rangatiratanga (ownership of Māori participation).	*Engagement*: Seeks to offer guidelines for the maternal‐infant healthcare system so that it can become culturally responsive partner for Māori whānau. The model was developed directly from Māori whānau experiences of the maternal‐infant healthcare system and is informed by Te Tiriti o Waitangi.	Manaakitanga (protecting and uplifting mana) Whakawhanaungatanga (relationships with people and space) Rangatiratanga (ownership of Māori participation).
Te Kapunga Putohe^5^	Restless hands is used to explain how nurses and health practitioners can work with Māori by showing how the hands can come together.	*Engagement*: Explains the culturally appropriate and acceptable ways interactions required for Māori‐centred practice, which keeps Māori and whānau at the centre to optimise efficacy when working with Māori.	Tikanga (rule, plan) Pono (being true and genuine) Aroha (compassion) Tiakitanga (being watchful) Manaakitanga (respect, kindness and hospitality) Tikanga Māori (Māori customs and practices) Mana tangata (personal authority) Oranga (wellbeing) Wairuatanga (spirituality) Whanaungatanga (connections)
Te Punga Oranga^6^	The growth of a fern is used to symbolize interrelated dimensions of health and wellbeing.	*Health and wellbeing*: The journey of the growth of a fern is used to symbolize the wellbeing journey of a person. Starting with the seeds that are planted and the consistent nourishment they need to grow, to the progression of the young shoots into a thriving fully grown fern. Each rau or leaf of the fern represents different dimensions of wellbeing that need to be nourished and grown to achieve overall health and wellbeing for Māori.	Pāpori (Social) Taiao (Environmental) Mahi (Occupational) Tinana (Physical) Aronganui (Emotional) Whānau and Iwi (Family and Community) Hinengaro (Mental) WaiTrua (Spiritual)
Te Whare Tapa Wha^7^	Four‐sided whare (house) to represents the four dimensions of health and wellbeing walls that need to be in balance.	*Health and wellbeing*: Te Whare Tapa reinforces the importance of all aspects of health and wellbeing being in balance. If one or more walls are not the same, it threatens the integrity of the house – that is the holistic health and wellbeing of the person and their whānau.	Tinana (body) Wairua (spirit) Whānau (extended family network) Hinengaro (mind)
Te Wheke^8,9^	The octopus is used to illustrate the multi‐dimensional aspect of health and wellbeing.	*Health and wellbeing*: Te Wheke's eyes show the person's wellbeing status, while the tentacles demonstrate the interrelatedness of the various inter‐related dimensions of health to explain the complexity of wellbeing for Māori.	Wairuatanga (spirituality) Mana ake (unique identity) Mauri (life force) Whanaungatanga (extended family) Tïnana (physical wellbeing) Hinengaro (the mind) Whatumanawa (open, healthy emotions) Whānau (family) Waiora (total wellbeing) Hā ā koro mā ā kuia mā (breath of life from ancestors)
Te Whetu^10^	The five‐pointed star is used to show the connectedness of all five components of Māori health and wellbeing.	*Health and wellbeing*: Highlights the need for understanding what are the important components for the health and wellbeing of Māori.	Whānau/whakapapa (family/genealogy) Whenua (land) Wairua (spirit) Hinengaro (mind) Tinana (body)

Note.

Note. ^1^Lacey et al., (2011); ^2^Robinson et al., (2020); ^3^Pitama et al., (2007); ^4^Stevenson (2018); ^5^Barton and Wilson (2008); ^6^Durie (1998); ^7^Murray (2010); ^8^Pere (1991), ^9^Love (2004); ^10^Mark and Lyons (2010).

**TABLE 2:** Summary of Key Aspects of the Māori Models of Health and Wellbeing

	Hui Process^1^	Kapakapa Manawa Framework^2^	Meihana Model^3^	Te Hā o Whānau^4^	Te Kapunga Putohe^5^	Te Punga Oranga^6^	Te Whare Tapa Wha^7^	Te Wheke^8,9^	Te Whetu^10^
*Dimensions of health and wellbeing*									
Wairua			⋆		⋆	⋆	⋆	⋆	⋆
Whānau	⋆		⋆	⋆	⋆	⋆	⋆	⋆	⋆
Hinengaro						⋆	⋆	⋆	
Tinana			⋆			⋆	⋆	⋆	⋆
*Whanaungatanga (Connections)*									
Whakapapa	⋆							⋆	⋆
Whānau			⋆						⋆
Whenua			⋆						⋆
Whanaungatanga	⋆	⋆	⋆	⋆	⋆			⋆	⋆
*Whakawhanaungatanga (Building relationships)*									
Tikanga			⋆		⋆				
Aroha		⋆			⋆				
Manaakitanga		⋆		⋆	⋆				
Mana				⋆	⋆			⋆	
Mauri					⋆			⋆	
Pono					⋆				
Tiakitanga					⋆				
*Socio‐political health context*									
Colonisation			⋆						⋆
Migration			⋆						
Racism			⋆						
Marginalisation			⋆						

Note.

^1^Lacey et al., (2011); ^2^Robinson et al., (2020); ^3^Pitama et al., (2007); ^4^Stevenson (2018); ^5^Barton and Wilson (2008); ^6^Durie (1998); ^7^Murray (2010); ^8^Pere (1991), ^9^Love (2004); ^10^Mark and Lyons (2010).

**TABLE 3:** Narrative overview of the results

Theme	Key elements	Description
Dimensions of health and wellbeing	Dimensions: Wairua (spiritual) Whānau (extended family network) Hinengaro (the mind) Tinana (physical)	Dimensions are interrelated and connected Wellbeing of person and whānau depends on dimensions being in balance Conceptualisations of wairua and beliefs influence engagement with healthcare practitioners Whānau are important and have collective roles and responsibilities for each other Hinengaro houses invisible, private thoughts and emotions Cultural protocols are important for caring for Māori and their whānau
Whanaungatanga (Connectedness)	Types of connections: Whakapapa (genealogy) Whenua (tribal and familial land) Whānau (immediate and extended family) Whanaungatanga (relational)	Past, present and future are inseparably connected For Māori, connectedness is dynamic Whānau is the primary mechanism for connectedness Remaining connected is vital for health and wellbeing Connection also includes those Māori have to mountains, rivers, and other key landmarks Whenua is a place to belong and stand in the world
Whakawhanaungatanga (Building relationships)	Requires: Establishing and maintaining relationships Building trust Aroha (empathy and compassion) and manaakitanga (kindness, generosity, caring for others) Mana‐enhancing (enhancing a person's status, power and authority) interactions	Face to face interactions are imperative Involves mutual and reciprocal sharing of information about who you are, where you are from and what you do Involves genuine, non‐judgmental attitudes and behaviours Supporting cultural customs and practices Equitable power‐sharing Involves offering cultural support and advocacy Responsibility to act in ways that demonstrate manaakitanga
Socio‐political health context	Colonisation Migration Racism Marginalisation	Socio‐political health context influences Māori health Healthcare practitioners are kaitiaki (guardians) of Māori and their whānau Effective interactions require exploring issues that impact Māori health and wellbeing Consider how the political environment marginalises Māori and their whānau. Be awareness of and resist using deficit stereotypes

### 3.1 Dimensions of holistic health and wellbeing

**FIGURE 2:**
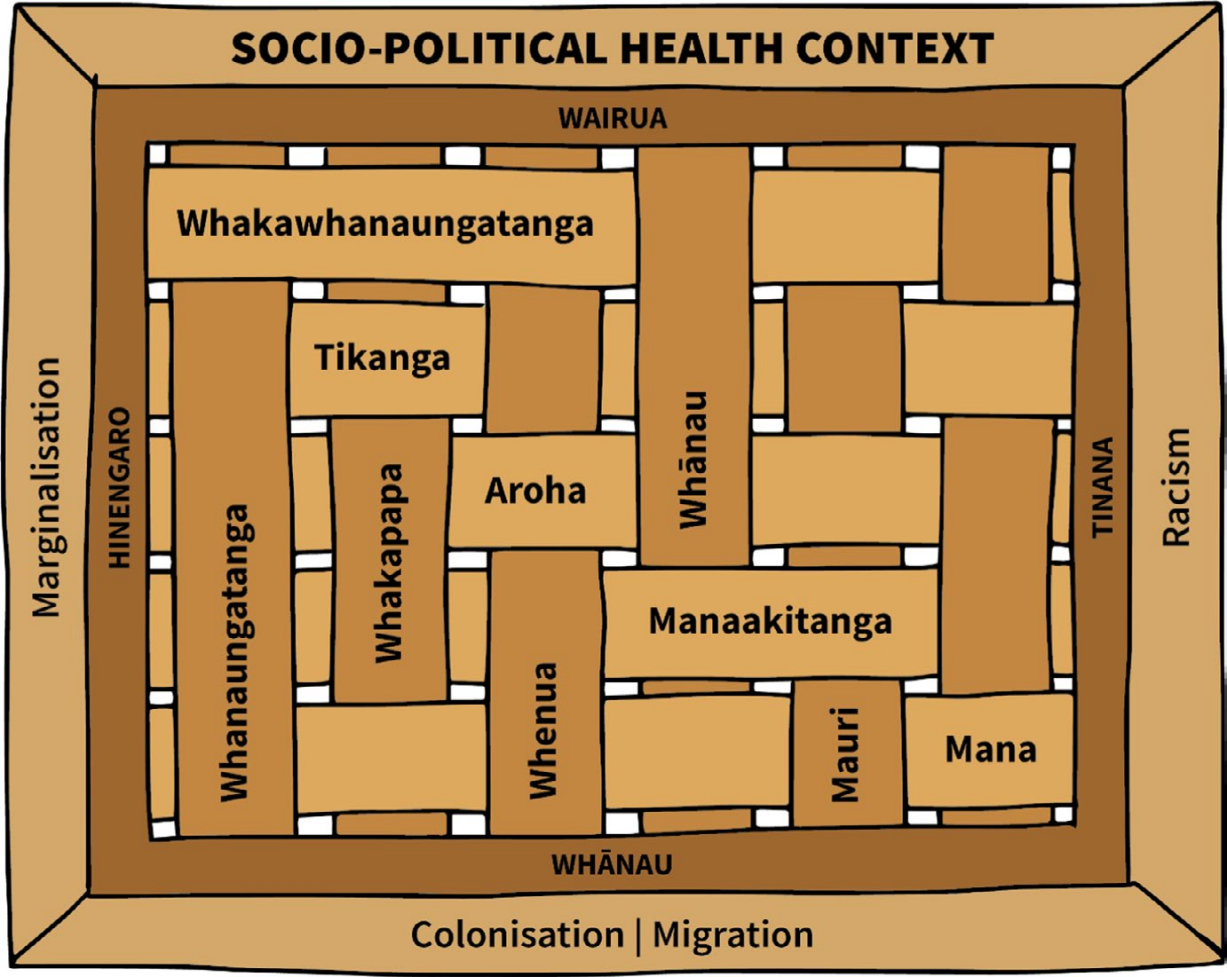
Overview of the themes arising from the literature review

The predominant dimensions of Māori holistic health and wellbeing found in the literature are wairua (spiritual), whānau (extended family network), hinengaro (the mind), and tinana (physical) (Barton & Wilson, 2008; Durie, 1998; Love, 2004; Mark & Lyons, 2010; Murray, 2010; Pere, 1991; Pitama et al., 2007; Stevenson, 2018). These dimensions of Māori health and wellbeing are interrelated and connected (see Figure 2). They are fundamental to a person's holistic wellbeing – a stark contrast from most predominant Western approaches to health that tend to view health in terms of physical wellbeing alone (Durie, 1998). Wairua, whānau, hinengaro and tinana are fundamentally interrelated, with the wellbeing of a person and their whānau reliant on all these dimensions being in balance.

Wairua, an often‐overlooked dimension, appeared in six of the Māori health and wellbeing models (Barton & Wilson, 2008; Durie, 1998; Love, 2004; Mark & Lyons, 2010; Murray, 2010; Pere, 1991; Pitama et al., 2007). Wairua refers to a person's spirit or soul that exists before the birth of a person and beyond their death. A person's wairua acts as a guide, warning of impending danger through visions and dreams. Importantly, wairua can be either protected or damaged (Mark & Lyons, 2010). The Meihana Model highlights how recognising and respecting a person's beliefs, values, and priorities when they engage with health services and health practitioners influence their state of wairua (Pitama et al., 2007). Māori healers interviewed during the Te Whetu model's development indicated wairua manifests in different ways, such as communicating with ancestors or receiving spiritual signs through animals, dreams and smells (Mark & Lyons, 2010).

Beliefs influence how Māori and whānau engage with health services. Te Kapunga Putohe highlights the importance of considering how a person conceptualises wairua to determine how their beliefs influence engagement with their health providers (Barton & Wilson, 2008). The Meihana Model suggests exploring a person's wairua by respectfully enquiring about spiritual and religious beliefs, special attachments to people, places, taonga (treasured items), and their beliefs about death and dying (Pitama et al., 2007). In these ways, healthcare practitioners can respectfully acknowledge and support the maintenance of a person's wairua and their whānau by joining them in karakia (Māori prayers) at meaningful times in their treatment journey, for instance.

Seven Māori health and wellbeing models highlighted the importance of whānau (Barton & Wilson, 2008; Durie, 1998; Love, 2004; Mark & Lyons, 2010; Murray, 2010; Pere, 1991; Pitama et al., 2007; Stevenson, 2018). Whānau describes the extended family network of Māori that is inclusive of a person's immediate and extended family. Whanau exemplifies the collective way in which Māori operate, and highlights how Māori do not exist in isolation of their collective whānau and community (Barton & Wilson, 2008; Durie, 1998; Love, 2004; Mark & Lyons, 2010; Murray, 2010; Pere, 1991; Pitama et al., 2007; Stevenson, 2018). Within the healthcare context, the Meihana model illustrates the collective roles and responsibilities Māori have in te ao Māori (the Māori world), particularly obligations individuals have to others within their whānau and the whānau as a whole entity (Pitama et al., 2007). This collective orientation highlights the imperative that healthcare practitioners include whānau as a person's support network, especially as whānau can also offer valuable social and medical history for a person. It is also important that healthcare practitioners assess whānau understanding of a patient's condition and provide appropriate education and support to whānau to ensure effective management of a whānau member's health.

Hinengaro featured in three Māori health and wellbeing models (Durie, 1998; Murray, 2010; Pere, 1991). Hinengaro refers to a person's mental dimension, which determines the expression of a person's feelings, sense of self, confidence, and self‐esteem. Central to hinengaro is mauri – the spark or essence of life – a state of potential that can be negatively or positively influenced by the environment (Durie, 1998). Hinengaro is the hidden female element within all people (Love, 2004) and involves the source of intuitive intelligence and senses (Pere, 1991). Hinengaro houses our invisible, private thoughts and emotions, indicating the caution required when questioning Māori with an expectation they will reveal their inner personal thoughts and feelings (Durie, 1998). For Māori, the use of indirect questions and metaphors might be more appropriate to avoid breaching a person's sense of privacy (Love, 2004).

Tinana refers to a person's physical dimension (Durie, 1998; Love, 2004; Mark & Lyons, 2010; Pere, 1991; Pitama et al., 2007). Tinana is respected as the body intricately linked to whānau and whakapapa. In particular, the role of women nurturing tamariki (children) from the womb until they reach the age to care for themselves (Pere, 1991). The tinana offers shelter from the external environment providing support for a person's essence (Love, 2004). Thus, the tinana of a person is more than a mere shelter for a person's wairua and hinengaro; it is also the source of sustenance for the person's body and health, regarded as being sacred (tapu).

Each of the models reviewed contained a core set of values, depending on the model's focus, whether it is about health and wellbeing or engagement with health services (see Table 2).

### 3.2 Whaunaungatanga (connectedness)

Māori health and wellbeing models are grounded in relationships and the notion of whanaungatanga or connectedness (see Figure 2). A sense of connectedness is vital for Māori, with whakapapa (genealogical connections), whenua (connections to tribal and familial land), and whānau (connections to both immediate and extended family) (Barton & Wilson, 2008; Durie, 1998; Love, 2004; Mark & Lyons, 2010; Murray, 2010; Pere, 1991; Pitama et al., 2007; Theunissen, 2011). Whanaungatanga conceptualises relational aspects of Māori culture. The expression and honouring of whanaungatanga occurs in numerous ways within te ao Māori, primarily through the process of whakawhanaungatanga – the establishment and maintenance of relationships.

The foundation for whanaungatanga is whakapapa, which is the process of tracing genealogical connections through the generations of a whānau to common ancestors. Whakapapa is exemplified by the whakatauakī included in the Te Wheke model, ‘*Hā ā koro mā*, *ā kui mā*’ (the breath of life from ancestors) (Pere, 1991). It affords Māori rights to whanaungatanga and the supports whānau can offer (Love, 2004; Mark & Lyons, 2010; Pere, 1991). Whakapapa provides continuity and unity between individuals, whānau, and hapū in the present, with their ancestors who have gone before them and nurturing future generations (Love, 2004; Pere, 1991). In these ways, the inextricable connection of the past, present, and future occur. A living generation represents the eternal breath of life passed from one generation to another, traced through whakapapa. In this way, whakapapa is evident in how Māori whānau are dynamically connected and interact to strengthen the whole, receiving ‘sustenance’ in the process. Being able to contribute to this process is essential for individuals’ health and wellbeing and their whānau. It is a source of identity, support, and comfort (Pere, 1991).

The dimension of whānau, as discussed previously in Dimensions of Holistic Health and Wellbeing, is closely entwined with and emerge out of whakapapa. Its inclusion here is because connections and connectedness are fundamental to Māori culture. Whānau is the primary mechanism for connectedness, whereby individuals are integral to the collective whānau. Individuals have an integral obligation and responsibility to the other members and the collective whānau. Therefore, the ability to remain connected to whānau is vital in maintaining Māori health and wellbeing.

Whakapapa also establishes connections not only to people but also to place. Whenua refers to both the placenta and the ancestral lands of a whānau, hapū and iwi. It is whakapapa that connects whānau to their placenta and the land (Mark & Lyons, 2010; Pitama et al., 2007). Te Whetu illustrates how whenua is a fundamental part of Māori identity, existence, and health. Whenua becomes evident when Māori connect who they are to specific maunga (mountain), awa (river), tūrangawaewae (place to stand – such as marae) as part of their whakapapa (Mark & Lyons, 2010; Pitama et al., 2007). The Meihana Model positions whenua as a specific spiritual or genealogical connection between people and land (Pitama et al., 2007). Maintenance of this connection to the land is through whenua, the transmission of pūrākau (culturally‐based stories), and other cultural traditions across generations.

Healthcare practitioners can engage with a person's whakapapa and whenua. It is a critical component of Māori identity by asking where they are from and their whakapapa or genealogical connections instead of focussing on where they currently live. Discussions about a person and their connections to whenua provide an opportunity for healthcare practitioners to engage in whakawhanaungatanga (the process of developing a relationship) by sharing knowledge and links to their whenua as part of this process.

### 3.3 Whakawhanaungatanga (Building relationships)

Whanaungatanga (connections) is an overarching theme within the Māori models of health and wellbeing (see Figure 2). Whakawhanaungatanga, the process of establishing and maintaining relationships, is optimally undertaken as face to face interaction with a genuine, non‐judgmental attitude to build trust. Whakawhanaungatanga is paramount and is a priority as part of collaborative healthcare engagement and activities. Underpinned by whanaungatanga, it is interrelated with whānau and is an integral part of three health and wellbeing models (Barton & Wilson, 2008; Lacey et al., 2011; Pere, 1991). Whanaungatanga is a source of kinship, social roles, and bonds within and outside the whānau – connectedness is vital to building a trusting relationship (Love, 2004). These connections are crucial to the health and wellbeing of the individual and their whānau and engagement with health services and practitioners.

The Hui (to gather or meet) process reinforces the notion that whakawhanaungatanga for Māori is more in‐depth than merely building rapport (Lacey et al., 2011). The process of whanaungatanga requires healthcare practitioners to acquire an understanding of te ao Māori from which to access knowledge about relevant cultural values, experiences, and beliefs held by Māori and their whānau. Critically, whakawhanaungatanga not only requires the person and their whānau to share information about aspects of themselves, but it also requires that the healthcare practitioners disclose and share information.

Tikanga (Māori cultural principles, practices, and customs) govern whakawhanaungatanga, a relational process (Barton & Wilson, 2008; Pitama et al., 2007). More specifically, tikanga guides the process of engagement and a person's actions to ensure they are respectful of everyone's status and that power is shared equitably within that relational space (Barton & Wilson, 2008; Lacey et al., 2011; Pere, 1991; Pitama et al., 2007; Robinson et al., 2020; Stevenson, 2018). For instance, Te Kapunga Putohe provides a framework for healthcare practitioners to ensure cultural customs and practices are supported and encouraged within health services delivery. Offering appropriate cultural support and advocacy for Māori and their whānau is imperative (Barton & Wilson, 2008). Implementing the Meihana Model requires healthcare practitioners to become familiar with cultural practices for Māori and their whānau and to work with Māori about how to enact these alongside clinical investigations and practices (Pitama et al., 2007).

Two cultural values that inform how Māori interact with others are aroha (empathy and compassion) and manaakitanga (kindness, generosity, and support to look after others) (Barton & Wilson, 2008; Robinson et al., 2020). Te Kapunga Putohe explains how a compassionate and empathetic approach from healthcare practitioners nurtures the development of mutual trust and respect with Māori patients and whānau (Barton & Wilson, 2008). The Kapakapa Manawa framework urges ‘he ngākau aroha’ (a heart of love), which entails healthcare practitioners developing relationships that communicate care, compassion and empathy by being kind‐hearted and considering others (Robinson et al., 2020). In addition to engaging with Māori and their whānau in an empathetic and compassionate way, manaakitanga involves demonstrating hospitality, kindness, caring, generosity, and respect. Te Hā o Whānau states that manaakitanga means meeting obligations in ways that uplift others’ mana (status and esteem) (Stevenson, 2018). Te Kapunga Putohe stresses manaakitanga encompasses many things in te ao Māori that enhances the mana of everyone. Thus, healthcare practitioners must act in ways that demonstrate manaakitanga when interacting with Māori patients and whānau (Barton & Wilson, 2008).

Mana stems from manaakitanga and refers to the status, prestige, and authority of Māori, their whānau, and others involved (Barton & Wilson, 2008; Love, 2004; Pere, 1991). The power associated with mana should be thought of in terms of ‘empowerment’ instead of ‘power over’ (Love, 2004). Everybody is born with mana, and a person's unique mana is linked to their whanau's mana. Mana can be enhanced or diminished by how others act and the actions of individuals themselves or their whānau. Intact mana is crucial for a positive cultural identity (Love, 2004; Pere, 1991). Thus, mana impacts both individual and collective identity. Te Kapunga Putohe refers to ‘mana tangata’, a person's power from within, and relates to their power and authority around self‐determining what is best for oneself (Barton & Wilson, 2008). It is also an acknowledgement that individual Māori do not exist in isolation but are part of a whānau collective. Necessary for mana is a person's mauri or energy that brings to life and binds them to all things in the physical world (Love, 2004; Pere, 1991). When the force or energy of Māori becomes weakened, a disconnection occurs between a person's tinana and wairua (Love, 2004). Thus, how healthcare practitioners undertake manaakitanga enhances the mana and mauri of all of those involved (Barton & Wilson, 2008; Robinson et al., 2020; Stevenson, 2018).

Some of the concepts, such as whanaungatanga, have core cultural values embedded within them. For instance, whanaungatanga encompasses whakapapa, whenua, and whānau, and is inclusive of values like tikanga, aroha, manaakitanga, and mana. Therefore, cultural engagement in a face to face manner aligns with a relational approach. It involves a formal process of mihimihi (greeting), whanaungatanga (everyone establishing their connections), aroha and manaakitanga (compassionately caring for and looking after visitors by ensuring they are fed, for instance), and mana (upholding people's status, authority, and esteem). Manaakitanga or how well people are looked after manifests in the host's mana. Whānau observes and discuss in the community the quality of care applied to the healthcare setting, the quality of engagement and relationships between Māori, their whānau, and healthcare practitioners.

### 3.4 Socio‐political health context

The Meihana model makes explicit that the cultural and socio‐political health context can influence Māori health (Pitama et al., 2007). Socio‐political context also informed the development of the other eight Māori health models. Three additional key areas notably impact negatively on Māori experiences of the health system and their health outcomes: colonisation and migration, racism, and marginalisation (see Figure 2). Te Kapunga Putohe also highlights the need for healthcare practitioners to act as kaitiaki (guardians) as Māori and their whānau enter the unfamiliar environments of health services and advocate for their needs (Barton & Wilson, 2008).

Consideration of the socio‐political health context requires healthcare practitioners to:

1. Undertake an exploration of issues linked to colonisation impacting the health and wellbeing of Māori and their whānau. These impacts include poverty, socioeconomic status, employment conditions, education opportunities, quality of housing, and the financial ability to engage with the health system.
2. Consider how political environments and events either alienate or include Māori communities in developing and implementing health services and programmes to improve Māori health outcomes.
3. Become aware of and resist deficit stereotypes of Māori that contribute to bias in clinical decision‐making when caring for Māori patients (Pitama et al., 2014).

Recognising people's socio‐political health context also encourages healthcare practitioners to understand and explore Māori patients and their whānau experiences living in a racialised society. Understanding Māori and their whānau contexts include being acutely aware of the impacts of institutional, interpersonal, and internalised racism, and becoming knowledgeable about racism and its effects on the health and wellbeing of Māori and their whānau (Pitama et al., 2014). Marginalisation relates to access to determinants of health, access to health services and the quality of healthcare. Therefore, healthcare practitioners should possess current socio‐political health context that includes knowledge about Māori health, including incidence and prevalence of illness and disease, morbidity, and mortality rates for Māori about specific illnesses or conditions to inform clinical assessment and practice decisions to reduce the marginalisation of Māori within the health system.

## 4 DISCUSSION

This review aimed to identify the key concepts, principles and values embedded within Indigenous Māori models of health and wellbeing; and determine how these could inform the development of a Māori‐centred relational model of care. We included ten papers that described nine Māori health models. The thematic analysis yielded four themes: (1) Dimensions of health and wellbeing; (2) Whanaungatanga (connectedness); (3) Whakawhanaugatanga (building relationships); and (4) Socio‐political health context.

The colonisation of Indigenous peoples globally has disconnected them from the protective factors embedded in their cultural values, beliefs and practices, and disenfranchised them politically and economically (Mulholland & Tawhai, 2010). Indigenous peoples have gone from being relatively healthy and prosperous to living with inequitable social marginalisation, racism and health disparities compared to those residing in their respective countries (Wilson et al., 2018). Pitama et al. (2014) define colonisation as "… both historical and ongoing" and has resulted in "… the loss of land, political re‐organisation and dehumanisation of Māori patients and/or community" (p. 112). A consequence of colonisation and assimilation policies (such as urbanisation, banning of speaking te reo Māori) meant the internal migration of Māori away from their tribal lands and the protective support structures of whānau and hapū (whānau constellations) (Mulholland & Tawhai, 2010). In practical terms, healthcare practitioners need to be aware that considerable diversity exists in the degree of cultural connectedness of Māori and whānau. That is, some Māori may not have ready access to whānau support. The inclusion of these concepts within the socio‐political health context extends our understanding of contextual factors which is critically important when providing healthcare for Indigenous populations, currently absent within the Fundamentals of Care framework.

There is a real and urgent need to address the quality of engagement with Māori when they interact with healthcare services. Aspinall et al., (2020) highlight the rationale for better engagement with Māori:

The outcomes of ongoing ineffective and disrespectful interactions affect their [Māori] trust in those within healthcare services and reinforce their perceptions that health services are unfriendly, complex and challenging to navigate – such experiences lead to avoidance of health services in the future (Aspinall et al., 2020, p. 4)

The development of a Māori‐centred relational care model grounded in the cultural values and practice protocols of Māori would promote respectful authentic relationships underpinned by compassion and kindness that goes with looking after other people – crucial in Māori society. Relational care is a reciprocal process. Therefore, healthcare practitioners need to be aware that they need to engage in whakawhanaungatanga authentically – this means sharing where you are from, who you are and what you do, for example. Besides creating favourable engagement experiences with healthcare services, positive physiological and psychological benefits are derived from affirmative relationships (Reis & Gable, 2003). Thus, healthcare providers need to be mindful not just of the importance of establishing relationships, which is the focus of the Fundamentals of Care framework, but of the tikanga (the process of enacting cultural protocols in the right way) when working with Māori within the health setting.

The Māori models of health and wellbeing demonstrate the importance of understanding the differences in worldview and cultural orientation. Health is a socio‐cultural construct that begins its formation within the contexts of a person's whānau, and overtime is shaped and refined (Wilson, 2008). Significantly, a person's cultural context influences their conceptualisation of health. The integration of wairua, whanau, hinengaro and tinana deepen the meaning of the existing Fundamentals of Care framework concepts of relational, physical and psychosocial care within a Māori centred view. Despite the Fundamentals of Care framework's potential, the second Australasian ILC meeting held in Auckland in 2019 highlighted the shortcomings for Māori and other similar groups. This Māori‐centred model of relational health integrates tikanga and provides a framework to bring into the existing Fundamentals of Care framework.

Future development of the Fundamentals of Care framework can redress the absence of recognition of the importance of authentic partnerships informed by health equity that honours the autonomy and self‐determination of Māori when engaging with healthcare practitioners (Aspinall et al., 2020; Wilson et al., 2018). Culture counts! However, there is always a risk of over‐simplifying rich, complex and challenging cultural concepts in publications like this one, particularly for use within Western health systems driven by dominant cultural contexts. The risk of over‐simplifying cultural concepts heightens further when non‐Māori healthcare practitioners simplify these rich, often circular cultural concepts and use them in superficial and linear ways.

**FIGURE 3:**
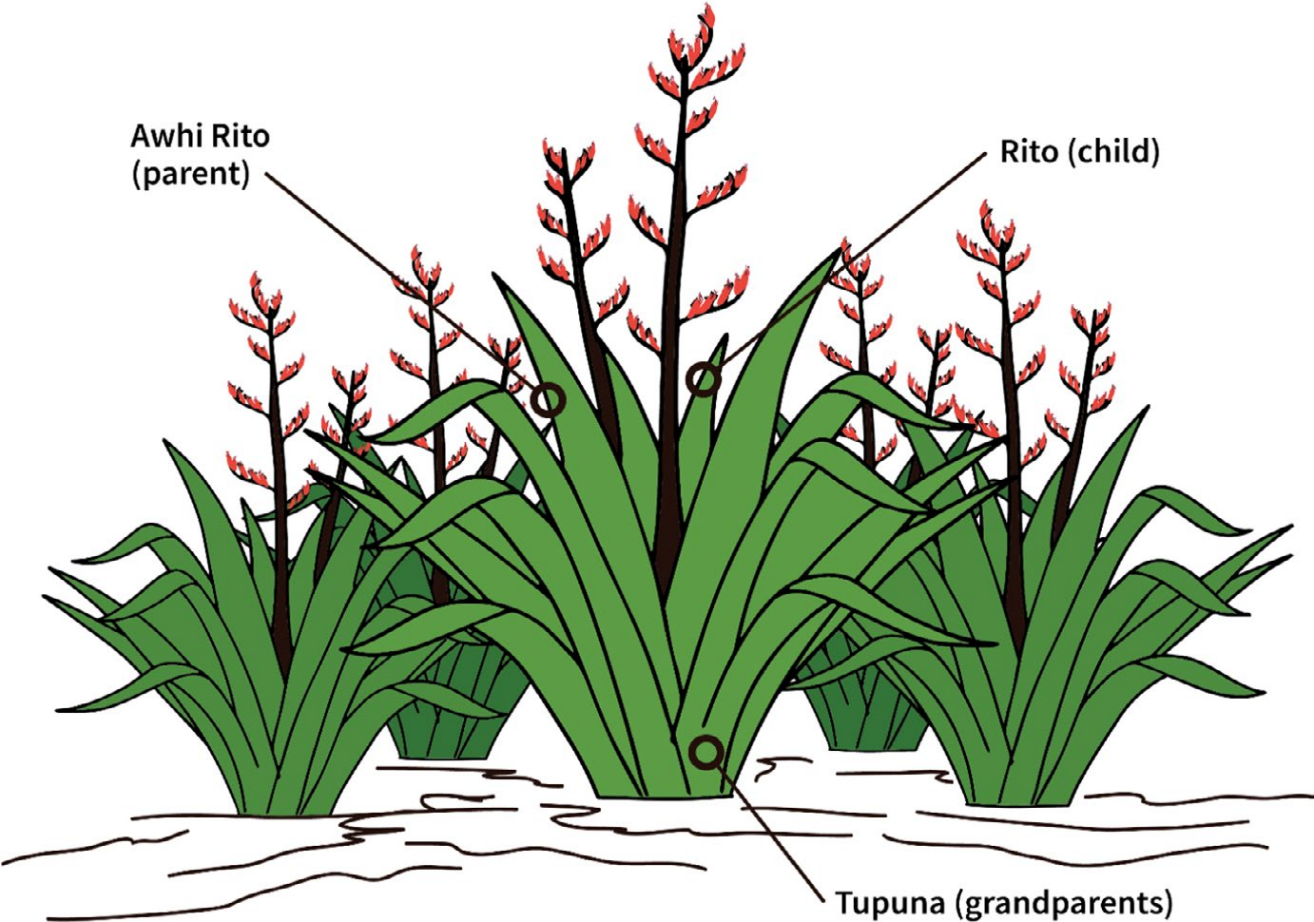
Pā Harakeke as a Metaphor for the Connectedness of Whānau

The *Pā Harakeke* (a grouping of native flax plants) is used as a metaphor to exemplify the multigenerational and functional importance of the connectedness of whānau for the collective wellbeing of the whānau and its members (see Figure 3), highlighted in the following whakataukī (proverb):

Hutia te rito o te harakeke, kei hea to komako e ko?

Ki te uia mai koe, he aha te mea nui o te ao?

Maku e ki atu, he tangata, he tangata, he tangata.

If you pull out the centre shoot of the flax plant, where will the bellbird sing?

If you ask me, what is the most important thing in the world?

I will tell you, it is people, it is people, it is people.

The rito (centre) shoot represents the tamariki (children) and is surrounded by the awhi rito, representing the parents, and the outer leaves that represent the tupuna (grandparents). The harakeke roots (whānau) provide the foundation and enable it to grow and connect to the surrounding harakeke for support. It is the collective strength of the whānau that ensures its members are cared for and protected. As the whakataukī above indicates, plucking the centre shoot threatens the wellbeing and integrity of the whānau. The Pā Harakeke highlights the importance of connectedness and whānau in the wellbeing of those seeking healthcare.

The depth of concepts embedded in Indigenous cultures draw on mātauranga (ancient and traditional Māori knowledge) observed and verified over centuries. For instance, hauora (holistic health and wellbeing) of Māori extends the understanding of health beyond individuals, problems, disease and illness. Therefore, we caution readers that this article is an introduction to integral cultural aspects associated with relational care for Indigenous Māori. Other Indigenous cultures will have similar but different holistic, relational views of health and wellbeing (Lambert et al., 2014).

For Māori, mātauranga Māori and tikanga provide essential signposts on the cultural imperatives when Māori engage with health services. Linked to adverse outcomes is the poor engagement of Māori and whānau seeking healthcare (Lacey et al., 2011; Wepa & Wilson, 2019). Healthcare practitioners knowledge of key mātauranga and tikanga associated with establishing relationships guides how to build better and meaningful relationships with Māori and their whānau to improve health outcomes and improve equity (Lyford & Cook, 2005; Pitama et al., 2007).

Active engagement with Māori and whānau is essential for establishing successful relationships and, importantly, improving Māori health equity. The processes of mihi (greeting) and whakawhanaungatanga are cultural imperatives for Māori, although the literature on Māori experiences of healthcare signal the need for healthcare practitioners to also be mindful about the manner with which they interact with Māori. Many Māori not trust health practitioners and health services because of their own or other whānau experiences. Therefore, engaging with Māori and whānau with genuine, caring and non‐judgmental attitudes is essential. Evidence supports Māori and whānau experiences of racism, discrimination and differences in the quality of care they receive (Cormack et al., 2018; Pitama et al., 2007; Rumball‐Smith et al., 2013; Wepa & Wilson, 2019; Wilson & Barton, 2012). Crucially, consideration of the treatment of Māori within health service contexts must occur given the detrimental impacts on health and wellbeing and the perpetuation of health inequities that adverse treatment and experiences can have for Māori and their whānau.

It is not unusual for Māori and whānau to feel culturally unsafe (Wepa & Wilson, 2019; Wilson & Barton, 2012). Aotearoa NZ has legislative obligations (Health Practitioners Competence Assurance Act (HPCA) 2003) requiring health practitioner regulatory bodies to ensure that all healthcare practitioners are culturally competent. In 2019, an amendment to section 118 (i) of the HPCA Act that replaced cultural competence wording with "cultural competence (including competencies that will enable effective and respectful interaction with Māori)." Generally, healthcare practitioners’ cultural competence is questionable with the inequities across the healthcare spectrum for Māori evident (Cormack et al., 2018; Health Quality and Safety Commission (HQSC), 2019). A lack of clarity and measurability exists across the regulated healthcare workforce in Aotearoa NZ that contribute to concerns about health care practitioners’ cultural competence and cultural safety for Māori and their whānau (Heke et al., 2018). A Māori‐centred relational model of care can provide healthcare practitioners with a practical way of respectful engagement with Māori necessary for effective interactions. It requires the inclusion of the dimensions of health and wellbeing for Māori, whanaungatanga, whakawhanaungatanga, and the socio‐political health context.

## 5 LIMITATIONS

This review did not include an appraisal for the quality of the papers included in this review (Grant & Booth, 2009) because instead, they were descriptions of Māori models of health rather than research reports. We only included English language papers, which we recognised may have lost the richness and depth in translating te reo Māori into English. There is the potential for bias even though we had two reviewers who independently undertook the analysis before engaging in consensus discussions to finalise the literature review's findings. To capture the essence of the cultural concepts that underpin a relational approach to care, we carried out an extensive cultural review process in writing this manuscript with Māori cultural advisors, fluent te reo Māori speakers, and experts in Māori health and healthcare service delivery to confirm the literature review findings.

## 6 CONCLUSION

While this literature review focussed on what was needed to inform a Māori‐centred relational model of care, it also shines a light on essential aspects for consideration. Figure 2 attempts to encapsulate the interconnectedness of various facets that impact the health and wellbeing of Māori and whānau. The dimensions of health care include the four pou or cornerstones that are interconnected; wairua (spirit), whānau (extended family network), hinengaro (mind), and tinana (body), although this is not an exclusive list. Whanaungatanga highlights how whakapapa, whenua and whānau form the foundations for connectedness for Māori. Together these provide the basis for whakawhanaungatanga. Building relationships, underpinned by tikanga and cultural values and concepts to ensure engagement with other people are mana‐enhancing, demonstrates that a Māori‐centred relational model of care is anything but basic and not a linear approach. Notably, a Māori‐centred model of relational care must also consider the socio‐political context that impacts Māori and their whānau and the healthcare that they receive.

## 7 FUNDING STATEMENT

No funding was received to prepare this article, including institutional support, non‐commercial grants, commercial support, and support‐in‐kind.

## ETHICAL REVIEW

Not required.

## AUTHORS’ CONTRIBUTIONS

EM and DW undertook the analysis, drafted, and revised the paper. JP organized engagement with Counties Manukau Health cultural advisors and Mana Whenua (tribal groups in the area that Counties Manukau Health serves), discussed the content and contributed to the writing of the manuscript. CA, and JS contributed to content discussions and the preparation of the manuscript.

## Supporting information

Supinfo

## Data Availability

Data were the 10 articles included for review.

## References

[jocn15859-bib-0001] Arksey, H. , & O'Malley, L. (2005). Scoping studies: Towards a methodological framework. International Journal of Social Research Methodology, 8(1), 19–32. 10.1080/1364557032000119616.

[jocn15859-bib-0002] Aspinall, C. , Parr, J. M. , Slark, J. , & Wilson, D. (2020). The culture conversation: Report from the 2nd Australasian ILC meeting—Auckland 2019. Journal of Clinical Nursing, 29(11‐12), 1768–1773. 10.1111/jocn.15281. 32279377

[jocn15859-bib-0003] Barton, P. , & Wilson, D. (2008). Te Kapunga Putohe (the restless hands): A Maori centred nursing practice model. Nursing Praxis of New Zealand, 24(2), 6–15. https://www.nursingpraxis.org/242‐te‐kapunga‐putohe‐the‐restless‐hands‐a‐maori‐centred‐nursing‐practice‐model.html. 18810900

[jocn15859-bib-0004] Cormack, D. , Stanley, J. , & Harris, R. (2018). Multiple forms of discrimination and relationships with health and wellbeing: Findings from national cross‐sectional surveys in Aotearoa/New Zealand. International Journal for Equity in Health, 17(1), 10.1186/s12939-018-0735-y. PMC5816516 29454356

[jocn15859-bib-0005] Davis, P. , Lay‐Yee, R. , Dyall, L. , Briant, R. , Sporle, A. , Brunt, D. , & Scott, A. (2006). Quality of hospital care for Māori patients in New Zealand: Retrospective cross‐sectional assessment. The Lancet, 367(9526), 1920–1925. 10.1016/s0140-6736(06)68847-8. 16765761

[jocn15859-bib-0006] Deeble, J. , Agar, J. S. , & Goss, J. (2008). Expenditure on health for Aboriginal and Torres Strait Islander peoples 2004–2005. Australian Institute of Health and Welfare. https://www.aihw.gov.au/reports/health‐welfare‐expenditure/expenditure‐health‐indigenous‐people‐2004‐05/formats.

[jocn15859-bib-0007] Durie, M. (1998). Whaiora: Maori health development, 2nd ed. Oxford University Press.

[jocn15859-bib-0008] Ellison‐Loschmann, L. , & Pearce, N. (2006). Improving access to health care among New Zealand’s Maori population. American Journal of Public Health, 96(4), 612–617. 10.2105/ajph.2005.070680. 16507721 PMC1470538

[jocn15859-bib-0052] Forsyth, H. (2017). Āta: An indigenous knowledge based pedagogical approach to teaching. Universal Journal of Educational Research, 5(10), 1729–1735. 10.13189/ujer.2017.051009.

[jocn15859-bib-0009] Gao, S. , Manns, B. J. , Culleton, B. F. , Tonelli, M. , Quan, H. , Crowshoe, L. , Ghali, W. A. , Svenson, L. W. , Ahmed, S. , & Hemmelgarn, B. R. (2008). Access to health care among status Aboriginal people with chronic kidney disease. Canadian Medical Association Journal, 179(10), 1007–1012. 10.1503/cmaj.080063. 18981441 PMC2572655

[jocn15859-bib-0010] Graham, R. , & Masters‐Awatere, B. (2020). Experiences of Māori of Aotearoa New Zealand's public health system: A systematic review of two decades of published qualitative research. Australian and New Zealand Journal of Public Health, 44(3), 193–200. 10.1111/1753-6405.12971. 32311187

[jocn15859-bib-0011] Grant, M. J. , & Booth, A. (2009). A typology of reviews: An analysis of 14 review types and associated methodologies. Health Information & Libraries Journal, 26(2), 91–108. 10.1111/j.1471-1842.2009.00848.x. 19490148

[jocn15859-bib-0012] Harris, R. , Cormack, D. , Tobias, M. , Yeh, L.‐C. , Talamaivao, N. , Minster, J. , & Timutimu, R. (2012a). The pervasive effects of racism: Experiences of racial discrimination in New Zealand over time and associations with multiple health domains. Social Science & Medicine, 74(3), 408–415. 10.1016/j.socscimed.2011.11.004. 22204840

[jocn15859-bib-0013] Harris, R. , Cormack, D. , Tobias, M. , Yeh, L‐C. , Talamaivao, N. , Minster, J. , & Timutimu, R. (2012). Self‐reported experience of racial discrimination and health care use in New Zealand: Results from the 2006/07 New Zealand Health Survey. American Journal of Public Health 102(5), 1012–1019. 10.2105/ajph.2011.300626. 22420811 PMC3483923

[jocn15859-bib-0014] Health Quality & Safety Commission (HQSC) . (2019). A window on the quality of Aotearoa New Zealand health care 2019. HQSC. https://www.hqsc.govt.nz/our‐programmes/health‐quality‐evaluation/publications‐and‐resources/publication/3721/.

[jocn15859-bib-0015] Heke, D. , Wilson, D. , & Came, H. (2018). Shades of competence? A critical analysis of the cultural competencies of the regulated‐health workforce in Aotearoa New Zealand. International Journal for Quality in Health Care. 10.1093/intqhc/mzy227. 30407524

[jocn15859-bib-0016] Jansen, P. , Bacal, K. , & Crengle, S. (2008). He Ritenga Whakaaro: Māori experiences of health services. Mauri Ora Associates. https://www.moh.govt.nz/NoteBook/nbbooks.nsf/0/2A6CAF401ABBEFB9CC2575F4000B6D0C?opendocument.

[jocn15859-bib-0017] Kingi, T. K. , Durie, M. , Elder, H. , Tapsell, R. , Lawrence, M. , & Bennett, S. (2017). Maea te toi ora: Māori health transformations. Huia Publishers.

[jocn15859-bib-0018] Kitson, A. L. (2018). The fundamentals of care framework as a point‐of‐care nursing theory. Nursing Research, 67(2), 99–107. 10.1097/nnr.0000000000000271. 29489631

[jocn15859-bib-0019] Kitson, A. L. , Muntlin Athlin, Å. , & Conroy, T. , (2014). Anything but basic: Nursing's challenge in meeting patients’ Fundamental Care Needs. Journal of Nursing Scholarship, 46(5), 331–339. 10.1111/jnu.12081. 24758508

[jocn15859-bib-0020] Kitson, A. , Conroy, T. , Kuluski, K. , Locock, L. , & Lyons, R. (2013). Reclaiming and redefining the fundamentals of care: Nursing's response to meeting patients’ basic human needs, 2nd ed. University of Adelaide. https://digital.library.adelaide.edu.au/dspace/bitstream/2440/75843/1/hdl_75843.pdf.

[jocn15859-bib-0021] Lacey, C. , Huria, T. , Beckert, L. , Gilles, M. , & Pitama, S. (2011). The Hui Process: A framework to enhance the doctor‐patient relationship with Māori. New Zealand Medcal Journal, 124(1347), 72–78. https://www.nzma.org.nz/journal‐articles/the‐hui‐process‐a‐framework‐to‐enhance‐the‐doctor‐patient‐relationship‐with‐maori. 22237570

[jocn15859-bib-0022] Lambert, M. , Luke, J. , Downey, B. , Crengle, S. , Kelaher, M. , Reid, S. , & Smylie, J. (2014). Health literacy: Health professionals’ understandings and their perceptions of barriers that Indigenous patients encounter. BMC Health Services Research, 14(1), 10.1186/s12913-014-0614-1. PMC4267746 25471387

[jocn15859-bib-0023] Love, C. (2004). Extensions on Te Wheke (Working Papers No. 6‐04). The Open Polytechnic of New Zealand. https://repository.openpolytechnic.ac.nz/handle/11072/182.

[jocn15859-bib-0024] Lyford, S. , & Cook, P. (2005). The Whanaungatanga model of care. Nursing Praxis in New Zealand, 21(2), 26–36. https://pubmed.ncbi.nlm.nih.gov/16764164/. 16764164

[jocn15859-bib-0025] Mark, G. T. , & Lyons, A. C. (2010). Maori healers’ views on wellbeing: The importance of mind, body, spirit, family and land. Social Science & Medicine, 70(11), 1756–1764. 10.1016/j.socscimed.2010.02.001. 20338680

[jocn15859-bib-0026] Ministry of Health (2015). Tatau Kahukura Māori health chart book 2015, 3rd ed. Ministry of Health. https://www.health.govt.nz/our‐work/populations/maori‐health/tatau‐kahukura‐maori‐health‐statistics.

[jocn15859-bib-0027] Mulholland, M. , & Tawhai, V. (2010). Weeping waters: The Treaty of Waitangi and constitutional change. Huia. https://huia.co.nz/huia‐bookshop/bookshop/weeping‐waters‐the‐treaty‐of‐waitangi‐and‐constitutional‐change/.

[jocn15859-bib-0028] Murray, L. (2010). Tau ora for our people. In S. Hoani , & S. Davis (Eds.), Toroa‐te‐Nukuroa Volume V: Ako Wānanga (pp. 112–117). https://ndhadeliver.natlib.govt.nz/delivery/DeliveryManagerServlet?dps_pid=IE6427401

[jocn15859-bib-0029] Parr, J. M , Bell, J. , & Koziol‐McLain, J. (2018). Evaluating fundamentals of care: The development of a unit‐level quality measurement and improvement programme. Journal of Clinical Nursing, 27(11‐12), 2360–2372. 10.1111/jocn.14250. 29292544

[jocn15859-bib-0030] Pere, R. R. (1991). Te wheke: A celebration of infinite wisdom. Ao Ako Global Learning. https://aoakogloballearning.co.nz/te‐wheke/.

[jocn15859-bib-0031] Pitama, S. , Robertson, P. , Cram, F. , Gillies, M. , Huria, T. , & Dallas‐Katoa, W. (2007). Meihana model: A clinical assessment framework. New Zealand Journal of Psychology, 36(3), 118–125. https://optforwellbeing.org/sites/default/files/events/Foundations/NZMJImprovingMaorihealththroughclinicalassessment1.pdf.

[jocn15859-bib-0032] Pohatu, T. W. (2013). Āta. Ata: Journal of Psychotherapy Aotearoa New Zealand, 17(1), 13–26. 10.9791/ajpanz.2013.02.

[jocn15859-bib-0033] Reis, H. T. , & Gable, S. L. (2003). Toward a positive psychology of relationships. In C. L. M. Keyes , & J. Haidt (Eds.), Flourishing: Positive psychology and the life well‐lived (pp. 129–159). American Psychological Association. https://www.apa.org/pubs/books/431686A.

[jocn15859-bib-0034] Robinson, J. , Moeke‐Maxell, T. , Parr, J. , Slark, J. , Black, S. , Williams, L. , & Gott, M. (2020). Optimising compassionate nursing care at the end of life in hospital settings. Journal of Clinical Nursing, 29(11‐12), 1788–1796. 10.1111/jocn.15050. 31495001

[jocn15859-bib-0035] Rumball‐Smith, J. , & Hider, P. (2009). The validity of readmission rate as a marker of the quality of hospital care, and a recommendation for its definition. New Zealand Medical Journal, 122(1289), 64–70. https://pubmed.ncbi.nlm.nih.gov/19305451/. 19305451

[jocn15859-bib-0036] Rumball‐Smith, J. , Sarfati, D. , Hider, P. , & Blakely, T. (2013). Ethnic disparities in the quality of hospital care in New Zealand, as measured by 30‐day rate of unplanned readmission/death. International Journal for Quality in Health Care, 25(3), 248–254. 10.1093/intqhc/mzt012. 23411833

[jocn15859-bib-0037] Sewell, J. (2017). Profiling the Māori health workforce 2017. Te Rau Matatini. Retrieved from https://terauora.com/wp‐content/uploads/2019/05/Profiling‐of‐the‐Ma%CC%84ori‐Health‐Workforce‐2017_Te‐Rau‐Matatini.pdf.

[jocn15859-bib-0039] Stevenson, K. (2018). Mā te wāhine, mā te whenua, ka ngaro te tangata. Wāhine and whānau experiences informing the maternal‐infant health care system. University of Otago. Retrievd from https://ourarchive.otago.ac.nz/handle/10523/8474

[jocn15859-bib-0040] Theunissen, K. E. (2011). The nurse's role in improving health disparities experienced by the indigenous Māori of New Zealand. Contemporary Nurse, 39(2), 281–286. 10.5172/conu.2011.281. 22551439

[jocn15859-bib-0041] Waitangi Tribunal (2019). Hauora ‐ Report on Stage One of the Health Services and Outcomes Kaupapa Inquiry: WAI 2575 Waitangi Tribunal Report 2019. Waitangi Tribunal. Retrieved from https://forms.justice.govt.nz/search/Documents/WT/wt_DOC_152801817/Hauora%20W.pdf.

[jocn15859-bib-0042] Walsh, M. , & Grey, C. (2019). The contribution of avoidable mortality to the life expectancy gap in Maori and Pacific populations in New Zealand‐a decomposition analysis. New Zealand Medical Journal, 132(1492), 46–60. https://www.nzma.org.nz/journal‐articles/the‐contribution‐of‐avoidable‐mortality‐to‐the‐life‐expectancy‐gap‐in‐maori‐and‐pacific‐populations‐in‐new‐zealand‐a‐decomposition‐analysis. 30921311

[jocn15859-bib-0043] Wepa, D. , & Wilson, D. (2019). Struggling to be involved: An interprofessional approach to examine Maori whanau engagement with healthcare services. Journal of Nursing Research and Practice, 03(03), 10.37532/jnrp.2019.3(3).1-5.

[jocn15859-bib-0044] Willams, P. N. , Gray, M. A. , Ka'ai, T. M. , Moorfield, J. C. , McPherson, K. M. , Weinstein, P. , & Nacey, J. N. (2003). Maori men's perceptions and experiences of health seeking for prostate health problems in New Zealand. Pacific Health Dialogue, 10(2), 71–78. https://pubmed.ncbi.nlm.nih.gov/18181419/. 18181419

[jocn15859-bib-0051] Wilson, D. (2008). The significance of a culturally appropriate health service for Indigenous Māori women. Contemporary Nurse, 28(1‐2), 173–188. 10.5172/conu.673.28.1-2.173. 18844571

[jocn15859-bib-0045] Wilson, D. , & Barton, P. (2012). Indigenous hospital experiences: A New Zealand case study. Journal of Clinical Nursing, 21(15–16), 2316–2326. 10.1111/j.1365-2702.2011.04042.x. 22642700

[jocn15859-bib-0046] Wilson, D. , & Haretuku, R. (2015). Te Tiriti o Waitangi/Treaty of Waitangi 1840: Its influence on health practice. In D. Wepa (Ed.), Cultural safety in Aotearoa New Zealand (pp. 79–99). Cambridge University Press. https://www.cambridge.org/highereducation/books/cultural‐safety‐in‐aotearoa‐new‐zealand/BCCD8C150F0A74889906C2241AB5AC46.

[jocn15859-bib-0047] Wilson, D. , Heaslip, V. , & Jackson, D. (2018). Improving equity and cultural responsiveness with marginalised communities: Understanding competing worldviews. Journal of Clinical Nursing, 27(19‐20), 3810–3819. 10.1111/jocn.14546. 29869819

[jocn15859-bib-0049] World Health Organization (2019). Social Determinants of Health. World Health Organization. Retrieved from https://www.who.int/health‐topics/social‐determinants‐of‐health#tab=tab_1.

[jocn15859-bib-0050] Zuckerman, S. , Haley, J. , Roubideaux, Y. , & Lillie‐Blanton, M. (2004). Health service access, use, and insurance coverage among American Indians/Alaska Natives and Whites: What role does the Indian Health Service play?. American Journal of Public Health, 94(1), 53–59. 10.2105/ajph.94.1.53. 14713698 PMC1449826

